# Comparative Evaluation of STOPPFrail and STOPP Criteria for Detecting Potentially Inappropriate Medications in Severely Frail Older Inpatients

**DOI:** 10.3390/healthcare14203433

**Published:** 2026-10-14

**Authors:** Yi-Chia Ku, Sheng-Yu Fan, Fang-Wen Hu, Jui-Fang Chen, Hui-Ning Tu, Chia-Ming Chang

**Affiliations:** 1 Department of Pharmacy, Tainan Municipal Hospital (Managed by Show Chwan Medical Care Corporation), Tainan 701033, Taiwan; 2 Institute of Gerontology, College of Medicine, National Cheng Kung University, Tainan 701401, Taiwan; 3 School of Nursing, Kaohsiung Medical University, Kaohsiung 807017, Taiwan; 4 Department of Pharmacy, National Cheng Kung University Hospital, Tainan 704302, Taiwan; 5 Department of Medicine, College of Medicine, National Cheng Kung University, Tainan 701401, Taiwan; 6 Department of Geriatrics and Gerontology, National Cheng Kung University Hospital, College of Medicine, National Cheng Kung University, Tainan 704302, Taiwan

**Keywords:** deprescribing, frail, polypharmacy, potentially inappropriate medications

## Abstract

**Highlights:**

**What are the main findings?**

• Potentially inappropriate medications (PIMs) were highly prevalent among severely frail older inpatients. STOPPFrail identified more PIMs than STOPP and mainly targeted preventive medications with limited expected benefit, whereas STOPP mainly targeted medications with immediate safety risks.
• Higher medication burden was associated with higher PIM counts under both criteria. STOPP-identified PIMs were additionally associated with female sex and fall history, whereas STOPPFrail-identified PIMs were associated with poorer nutritional status, admission through non-geriatric outpatient settings, and the CFS 9 subgroup, while facility residence and malignancy were associated with a lower burden of STOPPFrail-identified PIMs.

**What are the implications of the main findings?**

• The findings highlight differences in the PIM patterns identified by STOPPFrail and STOPP in severely frail older inpatients.
• Individualized medication assessment should consider patients’ clinical condition, prognosis, and goals of care.

**Abstract:**

Background: Severely frail older adults are highly vulnerable to potentially inappropriate medications (PIMs) due to multimorbidity and polypharmacy. Although the STOPP and STOPPFrail criteria were developed to identify PIMs in older patients, evidence comparing their utility in severely frail populations remains limited. This study aimed to compare PIMs identified by both criteria and to examine their associated clinical factors in severely frail older inpatients. Methods: This cross-sectional study enrolled 188 severely frail older inpatients (Clinical Frailty Scale ≥ 7) at a medical center in southern Taiwan. PIMs were identified using the STOPPFrail and STOPP criteria. Clinical variables collected included frailty status, cognitive function, nutritional status, comorbidity burden, disease complexity, history of falls, admission source, and number of medications. Results: STOPPFrail identified significantly more PIMs per patient than STOPP (mean of 2.2 vs. 1.4; *p* < 0.001) and detected at least one PIM in a greater proportion of patients (81.9% vs. 71.8%; *p* = 0.020). STOPPFrail predominantly flagged preventive medications, including antidiabetic agents, antihypertensives, lipid-lowering agents, and gastric acid suppressants, while STOPP more frequently identified sedatives, antipsychotics, antihypertensives, and anticoagulants. A higher number of regularly prescribed medications was associated with a higher PIM burden under both criteria. Female sex and a history of falls were additionally associated with STOPP-identified PIMs, whereas poorer nutritional status, admission from non-geriatric outpatient settings, and the CFS 9 subgroup were associated with a higher burden of STOPPFrail-identified PIMs, while facility residence and malignancy were associated with a lower burden. Conclusions: PIMs were highly prevalent among severely frail older inpatients. STOPPFrail primarily identified preventive medications with limited expected benefits, whereas STOPP focused on medications posing immediate safety risks. The two criteria sets identified different patterns of PIM use to guide individualized deprescribing in this vulnerable population.

## 1. Introduction

Frail older adults often experience multimorbidity and polypharmacy, which are associated with increased mortality, limited improvement in frailty status, and a higher risk of adverse hospital-related outcomes [1]. Recent multinational data indicate that polypharmacy is more prevalent in Asian countries, with particularly high levels observed in Taiwan [2]. In frail older adults, polypharmacy prevalence reaches up to 84%, while 31% experience persistent polypharmacy (≥181 days), reflecting a substantial and clinically relevant medication burden in routine practice [3].

Deprescribing is defined as the systematic process of discontinuing potentially inappropriate medications (PIMs) to mitigate drug-related problems and maintain function and quality of life [4]. Among those with severe frailty, these decisions can be particularly complex. Age-related pharmacokinetic changes, together with reduced physiological reserve in frailty, increase vulnerability to adverse drug effects and complicate medication management [5]. In addition, treatment goals in older adults with limited life expectancy may shift from disease prevention to symptom relief care, as preventive interventions may offer limited benefit when life expectancy is shorter than the time to benefit, and may increase treatment burden and potential harm [6,7,8]. Despite their clinical vulnerability, many patients do not receive systematic medication review until transition to palliative care, resulting in missed opportunities for earlier identification and discontinuation of PIMs [9]. Although deprescribing is often delayed in practice, emerging evidence from systematic reviews and meta-analyses suggests that deprescribing preventive medications in this population is not associated with increased risks of mortality or hospitalization. However, the certainty of evidence is very low, with heterogeneous findings possibly reflecting differences in study approaches, particularly the focus on individual preventive medications rather than standardized strategies [10].

In clinical practice, the Beers criteria [11] and STOPP (Screening Tool of Older Persons’ Prescriptions) [12] are widely used to identify PIMs associated with adverse drug events, including falls, delirium, and drug–disease interactions. However, these tools were not specifically developed for patients with advanced frailty or limited life expectancy and primarily focus on pharmacological safety. To address these gaps, the STOPPFrail criteria were developed to guide deprescribing in older adults with advanced frailty and limited life expectancy [13]. While STOPPFrail-guided interventions have been shown to reduce medication burden and are generally acceptable in nursing home and palliative care settings, their evidence base remains largely limited to these contexts, and their application in routine clinical practice remains limited [14,15,16].

Despite this, the STOPP and Beers criteria remain widely used in hospitalized older adults with severe frailty. Given the high medication burden in this population and lack of structured guidance, there is a need to clarify optimal medication review strategies. Compared with the Beers criteria, STOPP may have greater sensitivity in identifying PIMs associated with adverse clinical outcomes such as hospitalizations, supporting its use as a comparator to STOPPFrail [17].

This study therefore aimed to (1) compare the prevalence and categories of PIMs identified using the STOPPFrail versus STOPP criteria among hospitalized older adults with severe frailty, and (2) explore clinical factors associated with PIMs under each criterion. The findings may clarify how the two criteria differ in identifying inappropriate prescribing in advanced frailty.

## 2. Methods

### 2.1. Study Design and Participants

This cross-sectional study was conducted at a tertiary medical center in southern Taiwan (IRB number: B-ER-110-198). Participants were recruited from the geriatric and general internal medicine wards. A list of hospitalized patients was obtained from the hospital information system and screened twice weekly by a clinical pharmacist for eligibility. Hospitalized patients aged ≥65 years were eligible if they met all of the following criteria: Clinical Frailty Scale (CFS) score ≥ 7, indicating severe frailty [18]; poor one-year prognosis assessed using the “Surprise Question” by the attending physician (“Would I be surprised if this patient died within the next year?”) [19]; and polypharmacy, defined as ≥6 regularly prescribed medications. Patients were excluded if they had unstable clinical conditions, particularly hemodynamic instability (e.g., shock, cardiac arrest, or requiring vasopressor support). All participants provided written informed consent prior to enrollment. When a participant was judged unable to provide valid informed consent, written consent was instead obtained from the legally authorized representative. Participants were informed of the study purpose and procedures and signed consent forms before participation.

### 2.2. Data Collection and Measurements

Between July 2022 and November 2023, demographic and clinical information, including hospital wards (general internal medicine or geriatric medicine), sex, age, living condition (institution- or community-dwelling), utilization of home care or hospice home care services within the year prior to admission, primary diagnoses, and length of hospital stay, were collected at hospital admission. Pre-admission outpatient care was categorized as geriatric care (geriatric outpatient visits for >one month) versus usual care (geriatric outpatient visits for <one month or other specialty outpatient visits). Frailty status was assessed using the CFS, ranging from 1 (very fit) to 9 (terminally ill), with high inter-rater reliability (0.97) [18]. Nutritional status was evaluated using the Mini Nutritional Assessment Short-Form (MNA-SF), with scores < 7 indicating malnutrition [20]. Cognitive impairment was defined based on a documented diagnosis of dementia or cognitive decline. Delirium during hospitalization was assessed using the 3D Confusion Assessment Method (3D-CAM) [21]. Comorbidity burden was assessed using the Charlson Comorbidity Index (CCI) [22,23]. Disease complexity was evaluated using the Cumulative Illness Rating Scale-Geriatric (CIRS-G), which rates 14 organ systems from 0 (no problem) to 4 (extremely severe) [24]. Prognosis was estimated using the Prognostic Index, which incorporates factors including sex, activities of daily living (ADLs) dependency, heart failure, malignancy, renal dysfunction, and hypoalbuminemia. A score > 6 indicates a high-risk group associated with poor 1-year prognosis [25].

### 2.3. Medication Review and PIM Identification

Medication assessment was conducted by a trained clinical pharmacist with seven years of clinical experience in general medicine, using data from electronic health records and the national PharmaCloud System. PIMs were identified using the STOPPFrail (2021 version) [13] and STOPP (2015 version) criteria [12]. The pharmacist evaluated all regularly prescribed medications for duplication, dose appropriateness (based on liver and renal function within 48 h of admission), and potential major drug–drug interactions (severity “Major” with “Good” or “Excellent” evidence in Micromedex^®^). PIM identification was performed by the pharmacist, with judgments made in discussion with the attending physician based on clinical assessment. The number of chronic medications was defined as regularly prescribed medications taken continuously for at least 28 days, counting duplicate ingredients as a single medication. Medications prescribed outside our hospital that were covered by the National Health Insurance system were included; health supplements and self-paid medications were excluded. Pro re nata (PRN) medications were counted as regularly prescribed medications only if reported to have actually been taken on a regular basis for at least 28 days. The pharmacist also conducted clinical visits with the patient and primary caregiver to explore the patients’ medical conditions.

### 2.4. Statistical Analysis

Descriptive statistics were used to summarize the characteristics of the study population and the number of PIMs identified by the STOPP and STOPPFrail criteria. PIM prevalence was reported as the proportion of patients with at least one PIM identified by either the STOPP or STOPPFrail criteria. PIM categories were classified based on therapeutic drug classes as defined in each set of criteria. Although both tools include overlapping medication classes (e.g., antihypertensives or antidiabetic agents), the conditions under which they are considered potentially inappropriate differ between the two criteria, reflecting their distinct conceptual frameworks.

The normality of continuous variables was assessed via the Shapiro–Wilk test. McNemar’s test was used to compare the proportion of patients with ≥1 PIM identified by STOPP versus STOPPFrail. The Wilcoxon signed-rank test was used to compare the number of PIMs per patient, given the non-normal distribution of the data. The concordance between the STOPP and STOPPFrail criteria in identifying patients with ≥1 PIM was evaluated using Cohen’s κ statistic. Κ values were interpreted as follows: <0.4 indicating poor agreement, 0.4–0.6 moderate, 0.6–0.8 substantial, and >0.8 excellent.

Negative binomial regression was used to analyze PIM counts, with adjusted incidence rate ratios (IRRs), 95% confidence intervals, and *p*-values reported. Covariates were selected a priori based on their clinical relevance to frailty, comorbidity burden, and medication use: sex, facility residence, admission source, CFS, fall history, dementia, renal impairment, malignancy, age, MNA-SF, CIRS, and total number of regularly prescribed medications. Separate models were constructed for STOPP-PIM and STOPPFrail-PIM counts. The 12-covariate models were compared with models including all 20 candidate covariates using AIC and BIC (Supplementary Table S1). A sensitivity analysis excluding participants with CFS 9 was performed for both outcomes, with CFS 7 as the reference category (Supplementary Table S2). No correction for multiple comparisons was applied. A two-tailed *p*-value of <0.05 was considered statistically significant. All statistical analyses were conducted using IBM SPSS Statistics (version 22).

## 3. Results

### 3.1. Baseline Characteristics of the Study Participants

**Figure 1:**
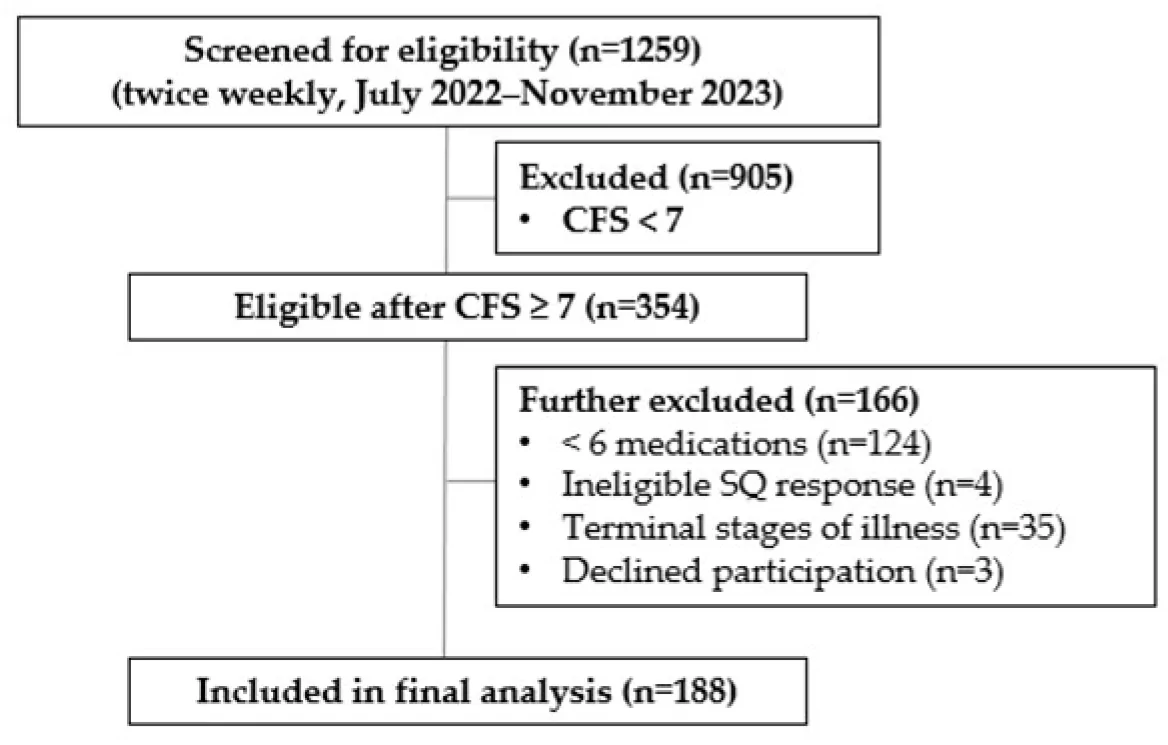
Re cruitment and participation. CFS: Clinical Frailty Scale; SQ: Surprise Question.

A total of 1259 patients were screened during the study period; 905 were excluded because their CFS values were <7. Of the remaining 354 patients, 166 were further excluded: 124 for taking fewer than 6 regularly prescribed medications, 4 for an ineligible Surprise Question response, 35 for unstable disease status, and 3 for declining to participate. The final analytic cohort comprised 188 patients (Figure 1).

The cohort comprised 188 severely frail inpatients (CFS ≥ 7) with high medication burden (10.7 ± 3.4 regular medications). Most were community-dwelling (67.6%), while 32.4% resided in long-term care facilities. During hospitalization, 75.5% experienced delirium, and 54.8% had a diagnosis of cognitive impairment or dementia. The mean MNA-SF score was below 7, indicating a high risk of malnutrition in this population. Regarding disease burden, the CCI averaged 7.40 and the CIRS-G averaged 2.83, indicating substantial comorbidity and disease complexity. The mean Prognostic Index (Walter et al. [25]) was 7.76 (median 7, IQR 6–9), and 124 participants (66.0%) had a score > 6, corresponding to a high-risk group for 1-year mortality in the original study. More than half (52.1%) of the patients required nasogastric tube feeding, and 41.5% had placement of urinary catheters, reflecting significant dependence on supportive medical care. The characteristics of the patients are presented in Table 1.

### 3.2. Discordance of PIMs Detected by STOPP and STOPPFrail Criteria

Table 2 shows a comparison of STOPP and STOPPFrail for detecting PIMs among participants. Of participants, 81.9% were identified as having ≥1 PIM identified by STOPPFrail criteria, compared with 71.8% by STOPP (*p* = 0.020). The mean number of PIMs per patient was significantly higher when identified by STOPPFrail (2.2 ± 1.9) than by STOPP (1.4 ± 1.3) (Z = −5.165, *p* < 0.001).

However, the agreement between the two criteria in identifying patients with ≥1 PIM was poor, with a Cohen’s κ coefficient of 0.101, indicating minimal concordance in classifying the same individuals as having PIMs due to divergent targeting. Based on the 2 × 2 classification counts in Table 2 (both positive: 114, STOPP-positive only: 21, STOPPFrail-positive only: 40, both negative: 13), the overall percent agreement was 67.6%, the positive percent agreement was 78.9%, and the negative percent agreement was 29.9%. The markedly higher positive than negative percent agreement indicates that the two tools agree more often when both identify a patient as having ≥1 PIM than when both identify a patient as having none, reflecting differing thresholds for a “negative” classification. The low κ is interpreted as evidence that the tools classify some patients differently, rather than demonstrating complementarity. STOPPFrail more frequently identified intensive-control medications with questionable long-term benefit–risk ratios in advanced frailty, such as antidiabetic agents in patients with HbA1c < 7.5% (25.0% vs. 11.2%, κ = 0.10), antihypertensives in patients with long-term BP control <130 mmHg (34.6% vs. 17.0%, κ = −0.028), and lipid-lowering agents in patients with low-density lipoprotein (LDL) < 70 (23.9% vs. 0). In contrast, STOPP more commonly targets medications associated with immediate pharmacologic safety risks, such as sedatives, antipsychotics, and anticoagulants. High inter-tool agreement occurred only in selected medication classes where safety and futility concerns overlap (e.g., antipsychotics, κ = 0.87; NSAIDs, κ = 0.87). After excluding the CFS 9 participants, STOPP and STOPPFrail identified PIMs in 71.3% and 81.8% of the remaining 181 participants, respectively (McNemar *p* = 0.019, Wilcoxon *p* < 0.001, κ = 0.107)—essentially unchanged from the full cohort (71.8% and 81.9%, κ = 0.101). The corresponding regression-based sensitivity analysis excluding CFS 9 is reported in Supplementary Table S2.

Table 3 compares the distribution of PIMs detected by STOPP and STOPPFrail across medication classes. Of the 2012 regularly prescribed medications reviewed, 12.9% were classified as PIMs by STOPP and 20.8% by STOPPFrail. STOPPFrail identified considerably more PIMs overall, with antihypertensives (27.4%) and antidiabetic agents (19.8%) being its most frequently flagged classes, reflecting its emphasis on avoiding potentially futile preventive therapies in severely frail patients. In contrast, STOPP’s largest category was sedatives (33.2%), highlighting its focus on medications posing immediate safety risks such as falls and cognitive impairment. While most shared medication classes were flagged more frequently by STOPPFrail, STOPP detected notably more PIMs involving antipsychotics (12.7% vs. 6.2%) and anticoagulants/antiplatelets (10.8% vs. 0.5%). Additionally, sedatives, antidepressants, and opioids were exclusive to STOPP, whereas lipid-lowering agents, calcium supplementation, memantine, and other preventive medications were flagged solely by STOPPFrail, underscoring the two tools’ complementary yet philosophically distinct approaches to identifying PIMs in older inpatients.

### 3.3. Associated Factors of PIMs Detected by STOPP and STOPPFrail Criteria

Table 4 compares the associated factors of PIMs detected by STOPP and STOPPFrail criteria. For STOPP-PIM, a higher expected PIM count was associated with female sex (IRR for male sex = 0.624, 95% CI 0.477–0.816; 95% CI 1.225–2.096, *p* = 0.001), fall history (IRR = 1.575, 95% CI 1.185–2.088, *p* = 0.002), and a higher number of regularly prescribed medications (IRR = 1.084, 95% CI 1.048–1.122, *p* < 0.001). Neither CFS 8 (IRR = 1.00, 95% CI 0.77–1.31, *p* = 0.997) nor CFS 9 (IRR = 0.82, 95% CI 0.41–1.65, *p* = 0.580) was significantly associated with STOPP-PIM.

For STOPPFrail-PIM, a higher expected PIM count was associated with CFS 9 (IRR = 2.002, 95% CI 1.099–3.647, *p* = 0.023), admission from non-geriatric outpatient settings (IRR = 1.678, 95% CI 1.135–2.481, *p* = 0.009), lower MNA-SF scores (IRR = 0.951, 95% CI 0.910–0.993, *p* = 0.023), and a higher number of regularly prescribed medications (IRR = 1.070, 95% CI 1.038–1.104, *p* < 0.001); by contrast, facility residence (IRR = 0.725, 95% CI 0.559–0.940, *p* = 0.015) and malignancy (IRR = 0.622, 95% CI 0.423–0.912, *p* = 0.015) were associated with a lower expected PIM count. CFS 8 alone was not significantly associated with STOPPFrail-PIM (IRR = 1.153, 95% CI 0.908–1.464, *p* = 0.242).

## 4. Discussion

This study provides a comparison of STOPP and STOPPFrail applications in hospitalized older adults with severe frailty. STOPPFrail identified significantly more PIMs than STOPP at the patient and medication levels, while concordance between the two tools was limited. Overall, both criteria identified high PIMs (STOPPFrail 81.9%, STOPP 71.8%). The STOPPFrail-PIM prevalence (81.9%) in our hospitalized cohort falls between nursing home reports (87.9%) [15] and palliative care settings (68.2%) [26]. This finding is consistent with previous studies showing that PIMs are frequently continued in patients with life-limiting illness, with deprescribing often delayed until late stages of care [9]. This pattern may reflect a clinical trajectory in which preventive medications are commonly maintained, while symptom-relief therapies are progressively introduced as disease advances, contributing to evolving medication burden [7,26,27]. In contrast, prescribing in palliative care tends to shift toward symptom-focused regimens, with increased use of pro re nata (PRN) medications and reduction in preventive medications [27]. Together, these findings underscore the need for earlier and more comprehensive medication review in patients with severe frailty.

In Table 2, the class-specific κ represents agreement in classification within a shared medication class, rather than agreement in the underlying clinical rationale. The discordance between the two tools reflects their fundamentally different development frameworks and clinical priorities. STOPP emphasizes pharmacological safety and more frequently identifies medications associated with adverse drug events, including sedatives (prolonged sedation, confusion, impaired balance, and increased fall risk), antihypertensives (e.g., bradycardia, electrolyte imbalance, and urinary incontinence), and anticoagulants (risk of bleeding). In contrast, STOPPFrail focuses on goal-concordant care by identifying preventive medications whose benefits are not aligned with limited prognosis and reduced physiological reserve in advanced frailty. For example, the cardiovascular benefits of lipid-lowering agents typically require >1–2 years to accrue, exceeding the prognosis of severely frail individuals. Intensive glycemic control with antidiabetic agents may increase the risk of hypoglycemia, outweighing benefits in advanced frailty. Aggressive blood pressure control increases orthostatic risk without clear mortality benefit in this population.

Across both models, a higher number of regularly prescribed medications was associated with higher PIM exposure. This finding should be interpreted cautiously because ≥6 regularly prescribed medications was an eligibility criterion. Although all participants met the study definition of polypharmacy, the medication count threshold alone does not fully capture the variation in PIM prescriptions. Table 4 shows that a greater number of regularly prescribed medications was associated with a higher number of PIM prescriptions. Thus, the observed association reflects variation in medication burden within a population that already met the polypharmacy criterion, rather than a dichotomous association between the presence of polypharmacy and PIMs.

For STOPP-PIMs, the presence of PIMs was significantly associated with polypharmacy, female sex, and fall history. Polypharmacy has been reported to be significantly associated with higher STOPP-PIMs [28]. A history of falls has also been linked to increased exposure to medications that elevate fall risk, including sedatives, antipsychotics, and certain antihypertensives [29]. Female sex was similarly associated with higher PIM exposure in prior studies, potentially contributing to an increased risk of adverse drug reactions in older women, partly related to greater use of prolonged benzodiazepines and antipsychotics, particularly among those with fall risk [30].

For STOPPFrail-PIMs, higher PIMs were associated with polypharmacy, admission from non-geriatric outpatient settings, malnutrition risk, and the CFS 9 subgroup. Patients admitted from non-geriatric outpatient settings showed higher STOPPFrail-PIMs, suggesting that patients admitted from geriatric outpatient settings may reflect prior medication optimization [31]. The association between malnutrition and STOPPFrail-PIM exposure is consistent with previous studies reporting that poorer nutritional status is associated with a higher prevalence of PIMs [32]. Geriatric syndromes, such as polypharmacy, malnutrition, falls, cognitive impairment, and depression, may occur simultaneously and interact with one another, resulting in deterioration (bidirectional association) and/or triggering the onset of other conditions [33]. Evidence from systematic reviews further suggests that deprescribing alone may have limited impact on outcomes such as falls or frailty, whereas more sustained benefits are observed when deprescribing is integrated into broader care models that include nutritional and functional support [34]. Notably, patients with malignant tumors and those residing in nursing homes had lower STOPPFrail-PIMs, consistent with prior findings that patients with life-limiting conditions are more likely to have preventive or limited-benefit medications deprescribed, although such changes are often concentrated near the transition to hospice care [9]. Patients in the CFS 9 subgroup had higher STOPPFrail-PIMs. This finding may partly reflect the use of glycemic and blood pressure thresholds in STOPPFrail to identify intensive treatment that may offer limited benefit in advanced frailty. However, given the small size of the CFS 9 subgroup (n = 7), this association should be interpreted cautiously and considered hypothesis-generating.

A medication classified as a PIM is not necessarily inappropriate for every individual; rather, the classification identifies a prescribing situation that warrants further clinical evaluation and individualized decision-making, and it should be interpreted together with clinical condition, prognosis, treatment goals, and patient preferences. Individualizing prescribing decisions according to remaining life expectancy provides an important conceptual framework for interpreting these differences [35], and STOPPFrail itself was specifically developed and validated for application in this end-of-life population [13]. Emerging pharmacist-led STOPPFrail intervention studies have begun to evaluate patient-centered outcomes such as medication burden, quality of life, falls, hospitalizations, and mortality [14], underscoring the need to move beyond PIM prevalence toward clinically meaningful outcomes. In clinical practice, we suggest that STOPP may serve as a broad screening tool during routine medication reconciliation and medication review, whereas STOPPFrail may provide additional information when prognosis is limited, during care transitions, or when goals of care become a central consideration. Regardless of the criteria used, medication decisions should integrate the patient’s clinical condition, prognosis, treatment goals, and preferences.

A methodological concern in this study relates to the use of the Surprise Question (SQ) to determine limited life expectancy eligibility for STOPPFrail application. While the SQ is widely used in clinical practice and has demonstrated a pooled prognostic accuracy of approximately 0.71 (95% CI 0.68 to 0.75) in systematic reviews, lower specificity and higher false-positive rates were reported in non-oncology inpatient populations [36]. As an inherently subjective, intuition-based instrument, the SQ is susceptible to inter-physician variability in prognostic judgment, which may have introduced heterogeneity in patient selection. Specifically, physicians with differing clinical experience, specialty backgrounds, or risk tolerance may have included patients without a genuinely limited life expectancy, potentially resulting in the inappropriate flagging of preventive medications that would otherwise be reasonable, thereby overestimating STOPPFrail-identified PIMs and affecting internal validity. Nevertheless, the original purpose of the SG is about early engagement of palliative care and supportive measures, e.g., deprescribing and symptom management, regardless of the mortality implications and predictive ability of the question diagnostically. Furthermore, the absence of a universally standardized method for determining limited life expectancy across the STOPPFrail literature—with prior studies employing varying approaches including frailty staging alone (CFS score ≥ 6), multidimensional geriatric assessment, or formal prognostic indices—limits direct comparability with previous studies. Future research would benefit from incorporating standardized, multi-component prognostic frameworks alongside the SQ to strengthen the rigor of patient selection and enhance cross-study reproducibility.

Overall, these findings indicate a high burden of PIMs in this population and highlight the need for a structured approach to deprescribing. Importantly, many individuals with advanced frailty remain community-dwelling and have not yet transitioned to institutional or palliative care settings. This suggests that medication review and identification of PIMs should not be deferred until end-of-life care, but instead initiated earlier in the frailty trajectory. STOPP and STOPPFrail identify different patterns of potentially inappropriate medication use for medication review; given that some STOPP criteria may be less applicable in functionally dependent individuals, their use requires individualized clinical judgment, and neither tool replaces individualized clinical judgment, shared decision-making, or patient/family preferences.

Several limitations should be considered when interpreting these findings. First, the single-center design may limit generalizability. Second, the inclusion criterion of polypharmacy (≥6 medications) may have selectively enrolled a higher-risk population with increased baseline PIM exposure, limiting generalizability, particularly in patients with prior medication optimization or deprescribing. As STOPP and STOPPFrail criteria are applied at the level of individual medications, requiring ≥6 regularly prescribed medications for eligibility likely contributed to the high observed prevalence of PIMs in this cohort. These findings may therefore not be generalizable to severely frail older adults taking fewer medications. Third, STOPP and STOPPFrail were applied in a cross-sectional manner, and therefore their impact on longitudinal clinical outcomes and their relative effectiveness in guiding deprescribing could not be evaluated. Fourth, while the STOPPFrail criteria explicitly consider limited life expectancy, prognosis in our cohort was estimated using the subjective “Surprise Question” posed to the attending physician. Although this approach is standard in palliative care identification, it may introduce variability or bias in applying the STOPPFrail criteria. This may have affected patient selection and comparability with previous studies. Fifth, PIM identification was performed by a single unblinded pharmacist. Although ambiguous all cases were discussed with the attending physician, inter-rater reliability was not evaluated. Potential observer or cross-classification bias may exist, particularly for criteria requiring clinical judgment. Sixth, given the sample size and number of covariates, overfitting cannot be completely excluded. Therefore, the multivariable findings should be interpreted as exploratory and hypothesis-generating, and *p*-values close to the conventional significance threshold should be interpreted cautiously. The analyses may still be underpowered for some comparisons. For example, estimates involving the small sample size (n = 7) in the CFS 9 subgroup need to be confirmed in larger studies. In addition, we cannot exclude the influence of additional unmeasured factors—such as prescribing culture, clinician specialty, and patient or family preferences—on prescribing patterns. Future studies should include multicenter designs to improve generalizability. Interventional studies comparing STOPP- and STOPPFrail-guided deprescribing are needed, evaluating patient-centered outcomes such as medication burden, adverse drug events, falls, functional status, quality of life, hospital readmission, mortality, caregiver burden, and successful discontinuation of PIMs. Further subgroup analyses by clinical specialty and care setting, as well as qualitative approaches exploring patient preferences, may help refine individualized deprescribing strategies in severely frail populations.

## 5. Conclusions

In conclusion, STOPPFrail identified more PIMs than STOPP (81.9% vs. 71.8%) among severely frail older inpatients, with minimal agreement between the two criteria. A higher number of regularly prescribed medications was associated with a higher PIM burden under both criteria; female sex and fall history were associated with STOPP-PIMs; poorer nutritional status, admission from non-geriatric outpatient settings, and the CFS 9 subgroup were associated with higher STOPPFrail-PIMs; and facility residence and malignancy were associated with lower STOPPFrail-PIMs. STOPP and STOPPFrail identified different patterns of PIM use for medication review in severely frail older adults. Further prospective studies are needed to determine whether criteria-guided deprescribing improves patient-centered clinical outcomes.

## Figures and Tables

**Table 1:** Baseline characteristics of the participants.

Variables	
Female	106 (56.4%)
Age, years	83.12 ± 8.04
Living condition	
Nursing home	61 (32.4%)
Community	127 (67.6%)
Palliative care prior to admission	10 (5.3%)
Pre-admission outpatient care	
Usual care	161 (85.6%)
Geriatric care	27 (14.4%)
Clinical Frailty Scale (CFS)	
CFS 7	86 (45.7%)
CFS 8	95 (50.5%)
CFS 9	7 (3.7%)
Placement of nasogastric tube	98 (52.1%)
Placement of urinary catheter	78 (41.5%)
Delirium	142 (75.5%)
Fall within a year	44 (23.4%)
Body mass index	21.21 ± 4.11
MNA-SF	6.67 ± 2.61
Charlson Comorbidity Index	7.40 ± 2.50
Prognostic index	7.76 ± 2.45
CIRS-G	2.83 ± 0.27
Diagnoses	
Myocardial infarction	14 (7.4%)
CVA	83 (44.1%)
Dementia	103 (54.8%)
Chronic heart failure	36 (19.1%)
COPD	27 (14.4%)
Chronic kidney disease	44 (23.4%)
Solid tumor	33 (17.6%)
Days of hospitalization	18.87 ± 11.54
Number of regularly prescribed medications	10.70 ± 3.35

Data are presented as number (%) of patients or mean ± standard deviation. Abbreviations: MNA-SF, Mini Nutritional Assessment Short-Form; CIRS-G, Cumulative Illness Rating Scale-Geriatric; CVA, cerebrovascular accident; COPD, chronic obstructive pulmonary disease.

**Table 2:** Comparison of STOPP and STOPPFrail for detecting PIMs among participants.

	STOPP-PIM (n, %)	STOPPFrail-PIM (n, %)	*p*-Value	Both (n) ^c^	Kappa ^d^
Overall patients with ≥1 PIM	135 (71.8%)	154 (81.9%)	0.020	114	0.101
PIMs per patient	1.38 ± 1.32	2.23 ± 1.93	<0.001	-	-
Antidiabetic agents	21 (11.2%)	47 (25.0%)	-	11	0.096
Antihypertensives	32 (17.0%)	65 (34.6%)	-	10	−0.028
Gastric acid suppressants	13 (6.9%)	36 (19.1%)	-	13	0.387
Antipsychotics	29 (15.4%)	23 (12.2%)	-	23	0.866
Systemic steroids	5 (2.7%)	23 (12.2%)	-	5	0.328
Nitrates	1 (0.5%)	13 (6.9%)	-	1	0.134
COPD agents	4 (2.1%)	10 (5.3%)	-	3	0.411
NSAIDs	8 (4.3%)	8 (4.3%)	-	7	0.869
Anticoagulants and antiplatelets	26 (13.8%)	2 (1.1%)	-	1	0.053
Sedatives ^a^	74 (39.4%)	-	-	-	-
Antidepressants ^a^	11 (5.9%)	-	-	-	-
Opioid agents ^a^	6 (3.2%)	-	-	-	-
Lipid-lowering agents ^b^	-	45 (23.9%)	-	-	-
Calcium supplements ^b^	-	18 (9.6%)	-	-	-
Memantine ^b^	-	14 (7.4%)	-	-	-
BPH agents ^b^	-	8 (4.3%)	-	-	-
Anti-resorptives/bone anabolics ^b^	-	4 (2.1%)	-	-	-
Antimuscarinic agents ^b^	-	3 (1.6%)	-	-	-

Abbreviations: COPD, chronic obstructive pulmonary disease; NSAID, nonsteroidal anti-inflammatory drug; BPH, benign prostatic hyperplasia. Each patient may have received more than one PIM. This table shows the number of patients affected per medication class. ^a^ STOPP only. ^b^ STOPPFrail only. ^c^ Identified by STOPP and STOPPFrail criteria. ^d^ Cohen’s κ is shown only for medication classes evaluated by STOPP and STOPPFrail; “-” marks a class evaluated by only one of the two tools, for which κ could not be calculated. Even for a class of medication evaluated by both tools, the two may apply different specific criteria when judging a medication as potentially inappropriate.

**Table 3:** Comparison of distribution of PIMs detected by STOPP and STOPPFrail across medication classes.

	STOPP-PIM	STOPPFrail-PIM
Classes of medications		
Antidiabetic agents	23 (8.9%)	83 (19.8%)
Antihypertensives	40 (15.4%)	115 (27.4%)
Gastric acid suppressants	13 (5.0%)	39 (9.3%)
Antipsychotics	33 (12.7%)	26 (6.2%)
Systemic steroids	5 (1.9%)	23 (5.5%)
Nitrates	1 (0.4%)	14 (3.3%)
COPD agents	4 (1.5%)	10 (2.4%)
NSAIDs	9 (3.5%)	9 (2.1%)
Anticoagulants and antiplatelets	28 (10.8%)	2 (0.5%)
Sedatives ^a^	86 (33.2%)	-
Antidepressants ^a^	11 (4.2%)	-
Opioid agents ^a^	6 (2.3%)	-
Lipid-lowering agents ^b^	-	51 (12.2%)
Calcium supplements ^b^	-	18 (4.3%)
Memantine ^b^	-	14 (3.3%)
BPH agents ^b^	-	8 (1.9%)
Anti-resorptives/bone anabolics ^b^	-	4 (1.0%)
Antimuscarinic agents ^b^	-	3 (0.7%)
Total (of 2012 medications)	259 (12.9%)	419 (20.8%)

^a^ STOPP only. ^b^ STOPPFrail only. Abbreviations: COPD, chronic obstructive pulmonary disease; NSAID, nonsteroidal anti-inflammatory drug; BPH, benign prostatic hyperplasia.

**Table 4:** Negative binomial regression model of PIMs detected by STOPP and STOPPFrail criteria.

Covariate	STOPP-PIM IRR (95% CI)	*p*	STOPPFrail-PIM IRR (95% CI)	*p*
Age	0.999 (0.983–1.016)	0.942	1.003 (0.989–1.018)	0.688
Gender (male)	0.624 (0.477–0.816)	0.001 *	1.050 (0.834–1.323)	0.676
Nursing home	1.041 (0.781–1.387)	0.787	0.725 (0.559–0.940)	0.015 *
Non-geriatric OPD admission	1.495 (0.964–2.315)	0.072	1.678 (1.135–2.481)	0.009 *
CFS 9	0.821 (0.408–1.651)	0.580	2.002 (1.099–3.647)	0.023 *
CFS 8	1.000 (0.765–1.306)	0.997	1.153 (0.908–1.464)	0.242
Fall within a year	1.575 (1.185–2.088)	0.002 *	0.873 (0.660–1.156)	0.344
Dementia	1.222 (0.940–1.592)	0.134	0.985 (0.779–1.247)	0.903
Chronic kidney disease	1.328 (0.989–1.783)	0.059	1.011 (0.770–1.330)	0.934
Solid tumor	1.058 (0.720–1.553)	0.775	0.622 (0.423–0.912)	0.015 *
MNA-SF	0.989 (0.940–1.041)	0.682	0.951 (0.910–0.993)	0.023 *
CIRS-G	1.286 (0.778–2.128)	0.326	0.780 (0.507–1.199)	0.257
Number of regularly prescribed medications	1.084 (1.048–1.122)	<0.001 *	1.070 (1.038–1.104)	<0.001 *

Abbreviations: CFS, Clinical Frailty Scale; MNA-SF, Mini Nutritional Assessment Short-Form; CIRS-G, Cumulative Illness Rating Scale-Geriatric; IRR, incidence rate ratio; CI: confidence interval. IRR > 1 indicates a higher expected PIM count and IRR < 1 indicates a lower expected count, relative to the reference category. * *p* < 0.05.

## Data Availability

The data of this study are available from the corresponding author upon reasonable request.

## Supplementary Materials

The following supporting information can be downloaded at https://www.mdpi.com/article/10.3390/healthcare14203433/s1, Table S1: Model fit of the 20- and 12-covariate models; Table S2: Sensitivity analysis excluding CFS 9 (*n* = 181); Table S3: STOPP and STOPPFrail criteria applied and number of medications meeting each criterion.
