# Supplementary material for: Comparative Evaluation of STOPPFrail and STOPP Criteria for Detecting Potentially Inappropriate Medications in Severely Frail Older Inpatients
Source: Healthcare (Basel). 2026 Oct 14;14(20):3433. doi: 10.3390/healthcare14203433 (PMC13704496; doi:10.3390/healthcare14203433)
Supplement: Supplementary file 1 [file healthcare-14-03433-s001.zip › healthcare-4554910-supplementary.pdf]

Supplementary Table S1. Model fit comparison (AIC/BIC) between the full 20-covariate model and the reduced 12-covariate model.

**Outcome**	**Model**	**AIC**	**BIC**
STOPP-PIM	20-covariate	563.570	638.008
STOPP-PIM	12-covariate	557.750	606.297
StoppFrail-PIM	20-covariate	708.626	783.064
StoppFrail-PIM	12-covariate	702.715	751.261

Supplementary Table S2. Sensitivity analysis excluding CFS 9 patients (n=181; CFS 8 vs. CFS 7 as reference).

**Covariate**	**STOPP-PIM IRR (95% CI)**	**P**	**StoppFrail-PIM IRR (95% CI)**	**P**
Gender (Male)	0.647 (0.493–0.850)	.002*	1.012 (0.801–1.279)	.919
Facility residence	1.009 (0.754–1.350)	.952	0.737 (0.568–0.956)	.022*
Admission source	1.490 (0.962–2.309)	.074	1.647 (1.117–2.425)	.012*
CFS 8 vs. CFS 7	1.015 (0.777–1.327)	.912	1.150 (0.907–1.458)	.250
Fall history	1.613 (1.206–2.158)	.001*	0.908 (0.680–1.213)	.513
Dementia	1.236 (0.943–1.623)	.126	0.958 (0.754–1.218)	.727
Renal impairment	1.315 (0.970–1.784)	.078	0.979 (0.739–1.297)	.883
Malignancy	1.069 (0.723–1.581)	.736	0.593 (0.396–0.888)	.011*
Age	0.997 (0.980–1.014)	.752	1.003 (0.989–1.018)	.672
MNA-SF	0.990 (0.940–1.043)	.706	0.957 (0.915–1.000)	.050*
CIRS	1.217 (0.730–2.027)	.451	0.850 (0.549–1.316)	.466
Total regular medications	1.087 (1.050–1.125)	<.001*	1.074 (1.042–1.107)	<.001*

Supplementary Table S3: List each STOPP and STOPPFrail criterion used, its operational definition, and the number of medications meeting each criterion, as appropriate.

Medication class	STOPP criteria	StoppFrail criteria
**Antidiabetic**	• Metformin if eGFR < 30 ml/min/1.73m^2 (risk of lactic acidosis). (n=10)<br>• Sulphonylureas with a long duration of action (e.g. glibenclamide, chlorpropamide, glimepiride) with type 2 diabetes mellitus (risk of prolonged hypoglycaemia). (n=10)<br>• Thiazolidenediones (e.g. rosiglitazone, pioglitazone) in patients with documented heart failure (risk of exacerbation of heart failure). (n=3)<br>**N = 23**	• Anti-diabetic drugs: De-intensify therapy. Avoid HbA1c targets (HbA1C <7.5% [58 mmol/mol] associated with net harm in this population). The goal of care is to minimise symptoms related to hyperglycaemia (e.g. excessive thirst, polyuria).<br>**N = 83**
**Antihypertensives**	• Verapamil or diltiazem with NYHA Class III or IV heart failure (may worsen heart failure). (n=2)<br>• Beta-blocker in combination with verapamil or diltiazem (risk of heart block). (n=0)<br>• Beta blocker with symptomatic bradycardia (< 50/min), type II heart block or complete heart block (risk of profound hypotension, asystole). (n=2)<br>• Loop diuretic as first-line treatment for hypertension (lack of outcome data for this indication; safer, more effective alternatives available). (n=0)<br>• Loop diuretic for dependent ankle oedema without clinical, biochemical evidence or radiological evidence of heart failure, liver failure, nephrotic syndrome or renal failure (leg elevation and/or compression hosiery usually more appropriate). (n=5)<br>• Thiazide diuretic with current significant hypokalaemia (i.e. serum K+ < 3.0 mmol/l), hyponatraemia (i.e. serum Na+ < 130 mmol/l), hypercalcaemia (i.e. corrected serum calcium > 2.65 mmol/l) or with a history of gout (hypokalaemia, hyponatraemia, hypercalcaemia and gout can be precipitated by thiazide diuretic). (n=6)<br>• Loop diuretic for treatment of hypertension with concurrent urinary incontinence (may exacerbate incontinence). (n=9)<br>• Centrally-acting antihypertensives (e.g. methyldopa, clonidine, moxonidine, rilmenidine, guanfacine), unless clear intolerance of, or lack of efficacy with, other classes of antihypertensives (centrally-active antihypertensives are generally less well tolerated by older people than younger people). (n=0)<br>• ACE inhibitors or Angiotensin Receptor Blockers in patients with hyperkalaemia. (n=12)<br>• Beta-blockers in diabetes mellitus with frequent hypoglycaemic episodes (risk of suppressing hypoglycaemic symptoms). (n=2)<br>• Vasodilator drugs (e.g. alpha-1 receptor blockers, calcium channel blockers, long-acting nitrates, ACE inhibitors, angiotensin I receptor blockers, diazoxide, minoxidil, hydralazine) with persistent postural hypotension i.e. recurrent drop in systolic blood pressure ≥ 20mmHg (risk of syncope, falls). (n=2)<br>**N = 40**	• Antihypertensive therapies: Carefully reduce or discontinue these drugs in patients with systolic blood pressure (SBP) persistently <130 mmHg. An appropriate SBP target in frail older people is 130–160 mmHg. Before stopping, consider whether the drug is treating additional conditions (e.g. beta-blocker for rate control in atrial fibrillation, diuretics for symptomatic heart failure).<br>N **= 115**
**Gastric acid suppressants**	• PPI for uncomplicated peptic ulcer disease or erosive peptic oesophagitis at full therapeutic dosage for > 8 weeks (dose reduction or earlier discontinuation indicated).<br>**N = 13**	• Proton pump Inhibitors: Reduce dose of proton pump inhibitors when used at full therapeutic dose ≥8 weeks, unless persistent dyspeptic symptoms at lower maintenance dose. (n=13)<br>• H2 receptor antagonist: Reduce dose of H2 receptor antagonists when used at full therapeutic dose for ≥8 weeks, unless persistent dyspeptic symptoms at lower maintenance dose. (n=26)<br>**N = 39**
**Antipsychotics**	• Neuroleptics with moderate-marked antimuscarinic/anticholinergic effects (chlorpromazine, clozapine, flupenthixol, fluphenzine, pipothiazine, promazine, zuclopenthixol)	• Neuroleptic antipsychotics in patients with dementia: Aim to reduce dose and discontinue these drugs in patients taking them for longer than 12 weeks if there are no current clinical features of

	with a history of prostatism or previous urinary retention (high risk of urinary retention). (n=3)<br>• Antipsychotics (i.e. other than quetiapine or clozapine) in those with parkinsonism or Lewy Body Disease (risk of severe extra-pyramidal symptoms). (n=4)<br>• Neuroleptic antipsychotic in patients with behavioural and psychological symptoms of dementia (BPSD) unless symptoms are severe and other treatments have failed (increased risk of stroke). (n=26)<br>**N = 33**	behavioural and psychiatric symptoms of dementia (BPSD).<br>**N = 26**
**Systemic steroids**	• Systemic corticosteroids instead of inhaled corticosteroids for maintenance therapy in moderate-severe COPD (unnecessary exposure to long-term side-effects of systemic corticosteroids and effective inhaled therapies are available). (n=4)<br>• Long-term corticosteroids (>3 months) as monotherapy for rheumatoid arthritis (risk of systemic corticosteroid side-effects). (n=1)<br>**N = 5**	• Long-term oral corticosteroids: Increased risk of major side effects (e.g. fragility fractures, proximal myopathy, peptic ulcer disease) when taken regularly for ≥2 months. Consider careful dose reduction and discontinuation.<br>**N = 23**
**Nitrates**	• Vasodilator drugs (e.g. alpha-1 receptor blockers, calcium channel blockers, long-acting nitrates, ACE inhibitors, angiotensin I receptor blockers, diazoxide, minoxidil, hydralazine) with persistent postural hypotension i.e. recurrent drop in systolic blood pressure ≥ 20mmHg (risk of syncope, falls).<br>**N = 1**	• Anti-anginal therapy (specifically nitrates, nicorandil, ranolazine): None of these anti-anginal drugs have been proven to reduce cardiovascular mortality or the rate of myocardial infarction. Aim to carefully reduce and discontinue these drugs in patients who have had no reported anginal symptoms in the previous 12 months AND who have no proven or objective evidence of coronary artery disease.<br>**N = 14**
**COPD agents COPD**	• Theophylline as monotherapy for COPD (safer, more effective alternative; risk of adverse effects due to narrow therapeutic index).<br>**N = 4**	• Theophylline and aminophylline: These drugs have a narrow therapeutic index, have doubtful therapeutic benefit and require monitoring of serum levels and interact with other commonly prescribed drugs putting patients at an increased risk of ADEs. (n=7)<br>• Leukotriene antagonists (montelukast, zafirlukast): These drugs have no proven role in chronic obstructive pulmonary disease; they are indicated only in asthma. (n=3)<br>**N = 10**
**NSAIDs**	• NSAID's if eGFR < 50 ml/min/1.73m^2 (risk of deterioration in renal function). (n=2)<br>• Non-COX-2 selective non-steroidal anti-inflammatory drug (NSAID) with history of peptic ulcer disease or gastrointestinal bleeding, unless with concurrent PPI or H2 antagonist (risk of peptic ulcer relapse). (n=1)<br>• NSAID with established hypertension (risk of exacerbation of hypertension) or heart failure (risk of exacerbation of heart failure). (n=2)<br>• Long-term use of NSAID (>3 months) for symptom relief of osteoarthritis pain where paracetamol has not been tried (simple analgesics preferable and usually as effective for pain relief). (n=1)<br>• COX-2 selective NSAIDs with concurrent cardiovascular disease (increased risk of myocardial infarction and stroke). (n=3)<br>**N = 9**	• Long-term oral nonsteroidal anti-inflammatory drugs: Increased risk of side effects (e.g. peptic ulcer disease, bleeding, worsening heart failure) when taken regularly for ≥2 months.<br>**N = 9**
**Anticoagula nt and antiplatelets**	• Aspirin with a past history of peptic ulcer disease without concomitant PPI (risk of recurrent peptic ulcer). (n=3)<br>• Aspirin, clopidogrel, dipyridamole, vitamin K antagonists, direct thrombin inhibitors or factor Xa inhibitors with concurrent significant bleeding risk, i.e. uncontrolled severe hypertension, bleeding diathesis, recent non-trivial spontaneous bleeding (high risk of bleeding). (n=23)<br>• Aspirin plus clopidogrel as secondary stroke prevention, unless the patient has a coronary stent(s) inserted in the previous 12 months or	• Anti-platelets: No evidence of benefit for primary (as distinct from secondary) cardiovascular prevention. (n=1)<br>• Aspirin for stroke prevention in atrial fibrillation: Aspirin has little or no role for stroke prevention in frail older people who are not candidates for anticoagulation therapy and may significantly increase bleeding risk. (n=1)<br>**N = 2**

	concurrent acute coronary syndrome or has a high grade symptomatic carotid arterial stenosis (no evidence of added benefit over clopidogrel monotherapy). (n=1)<br>• Ticlopidine in any circumstances (clopidogrel and prasugrel have similar efficacy, stronger evidence and fewer side-effects). (n=1)<br>**N = 28**	
**Sedatives**	• Benzodiazepines for ≥ 4 weeks (no indication for longer treatment; risk of prolonged sedation, confusion, impaired balance, falls, road traffic accidents; all benzodiazepines should be withdrawn gradually if taken for > 2 weeks as there is a risk of causing a benzodiazepine withdrawal syndrome if stopped abruptly). (n=58)<br>• Benzodiazepines with acute or chronic respiratory failure i.e. pO2 < 8.0 kPa ± pCO2 > 6.5 kPa (risk of exacerbation of respiratory failure). (n=20)<br>• Hypnotic Z-drugs (e.g. zopiclone, zolpidem, zaleplon) (may cause protracted daytime sedation, ataxia). (n=8)<br>**N = 86**	(no corresponding criterion)
**Antidepressants**	• Tricyclic antidepressants with dementia, narrow angle glaucoma, cardiac conduction abnormalities, prostatism, or prior history of urinary retention (risk of worsening these conditions). (n=7)<br>• Selective serotonin re-uptake inhibitors (SSRI's) with current or recent significant hyponatraemia i.e. serum Na+ < 130 mmol/l (risk of exacerbating or precipitating hyponatraemia). (n=4)<br>**N = 11**	(no corresponding criterion)
**Opioid agents**	• Use of oral or transdermal strong opioids (morphine, oxycodone, fentanyl, buprenorphine, diamorphine, methadone, tramadol, pethidine, pentazocine) as first line therapy for mild pain (WHO analgesic ladder not observed). (n=0)<br>• Use of regular (as distinct from PRN) opioids without concomitant laxative (risk of severe constipation). (n=6)<br>• Long-acting opioids without short-acting opioids for break-through pain (risk of non-control of severe pain). (n=0)<br>**N = 6**	(no corresponding criterion)
**Lipid lowering agents**	(no corresponding criterion)	• Lipid-lowering therapies (statins, ezetimibe, bile acid sequestrants, fibrates, nicotinic acid, lomitapide and acipimox).<br>**N = 51**
**Calcium supplements**	(no corresponding criterion)	• Calcium supplements: Unlikely to be of any benefit in short-term unless proven, symptomatic hypocalcaemia.<br>**N = 18**
**Memantine**	(no corresponding criterion)	• Memantine: Discontinue and monitor in patients with moderate to severe dementia, unless memantine has clearly improved BPSD.<br>**N = 14**
**BPH agents**	(no corresponding criterion)	• Drugs for benign prostatic hyperplasia (5-alpha reductase inhibitors and alpha-blockers) in catheterised male patients: No benefit with long-term bladder catheterisation.<br>**N = 8**
**Anti-resorptive/bone anabolic**	(no corresponding criterion)	• Anti-resorptive/bone anabolic drugs for osteoporosis (bisphosphonates, strontium, teriparatide, denosumab).<br>**N = 4**
**Antimuscarinic agents**	(no corresponding criterion)	• Drugs for overactive bladder (muscarinic antagonists and mirabegron): No benefit in patients with persistent, irreversible urinary incontinence unless clear history of painful detrusor hyperactivity.<br>**N = 3**
